# Resistance of breast cancer cells to paclitaxel is associated with low expressions of miRNA-186 and miRNA-7

**DOI:** 10.20517/cdr.2023.19

**Published:** 2023-09-01

**Authors:** Vera Apollonova, Daniil Plevako, Alexandr Garanin, Elena Sidina, Lidia Zabegina, Margarita Knyazeva, Viktoria Smirnova, Anna Artemyeva, Petr Krivorotko, Anastasia Malek

**Affiliations:** ^1^Breast Surgical Oncology, N.N. Petrov National Medical Research Center of Oncology, St. Petersburg 197758, Russia.; ^2^Subcellular Technology Lab, N.N. Petrov National Medical Research Center of Oncology, St. Petersburg 197758, Russia.; ^3^ Department of Pathology, N.N. Petrov National Medical Research Center of Oncology, St. Petersburg 197758, Russia.; ^#^Authors contributed equally.

**Keywords:** Breast cancer, paclitaxel, resistance, neoadjuvant therapy, miRNA, miR-186, miR-7

## Abstract

**Aim:** Neo-adjuvant chemotherapy is a common approach for the complex treatment of breast cancer (BC) and paclitaxel (PTX) is frequently included in the therapeutic regimen. However, the effect of PTX-based treatment is hard to predict precisely based on routinely used markers. As microRNAs are considered a new promising class of biomarkers, the link between miRNA expression and PTX resistance of BC cells needs to be well investigated. This study aimed at the identification of miRNAs associated with responses of BC cells to PTX.

**Methods:** Intrinsic PTX sensitivity and miRNA profiling were assayed in five BC cell lines to identify candidate miRNAs. Selected miRNA (n. 15) expressions were analyzed by real-time-quantitative polymerase chain reaction (RT-qPCR) in BC tissue samples (n. 31) obtained from a diagnostic biopsy. Results were analyzed in the context of the effect of two cycles of PTX and the effect of the completed scheme of neoadjuvant therapy. The study’s design facilitated the evaluation of the effect of PTX on cells and the identification of features of the microRNA expression profiles associated exclusively with sensitivity to this drug.

**Results:** miR-186 and miR-7 expression in BC tissues was higher in patients with better outcomes of PTX-based neoadjuvant therapy.

**Conclusion:** High expressions of miR-186 and miR-7 are associated with good response to PTX, whereas their low expressions may be associated with resistance to PTX in BC, indicating the possibility of developing innovative test systems for the prediction of the PTX response, which can be used before the start of neo-adjuvant chemotherapy for BC.

## INTRODUCTION

Breast cancer (BC) is a common oncological disease. According to the International Agency for Research on Cancer, more than 2.2 million women were diagnosed with BC in 2020^[1]^. Despite the constant progress in the development of new diagnostic and therapeutic approaches, BC poses a significant social burden across countries with different economies and healthcare systems. One of the most intriguing problems encountered in the treatment of BC is the disease’s extreme heterogeneity^[2]^, which makes it difficult to choose the optimal approach for systemic therapy. Different molecular markers including sex hormone receptors (ER, PR), receptor of epidermal growth factors (HER2), markers of active proliferation (Ki-67, survivin, NGAL), and metastatic potency (MMP9, SK1, DcR3, CIX2, EZH2) are used currently in clinical practice to evaluate tumor properties and define the optimal therapeutic approaches. Several new tests and classifiers have been developed recently, although not approved so far, including PAM50^[3]^, EndoPredict^[4]^, Oncotype DX, Breast Recurrence Score, EndoPredict, Prosigna^[5]^, and others. However, the selection of BC chemotherapy regimen in clinical practice is still based on the short list of tumor characteristics such as histology, grade, stage, and IHC status of hormone receptors (HR), HER2, and Ki-67^[6]^. These characteristics are far from exhaustive and their complex evaluation does not allow prediction of the inherent sensitivity of BC cells to specific cytostatic agents.

Neoadjuvant therapy (NAT) is integral to the treatment of patients with high-risk breast tumors, large breast tumors, and locally advanced tumors, including those who are initially ineligible for surgery. Several schemes of NAT are approved for early-stage BC of various biological types^[7]^. For instance, neoadjuvant chemotherapy of HER2-negative BC may include different combinations of cytostatic agents such as anthracyclines, cyclophosphamide, and taxanes^[7]^ that can be administered in different sequential orders^[8]^. These drugs have varied mechanisms of action-anthracyclines intercalate between DNA base pairs and block essential processes of DNA transcription and replication^[9]^; cyclophosphamide is an alkylating agent forming intrastrand and interstrand DNA cross-links, thereby affecting DNA function^[10]^, whereas taxanes stabilize microtubules and affect the dynamic of the mitotic spindle^[11]^. In the background of different mechanisms of action of these drugs, differential intrinsic sensitivity of an individual tumor to each of these is expected. Initiating NAT with presumably the most effective drug or their combination would provide the best response; however, new molecular markers are required to personalize the selection of optimal therapeutic scheme from a list of validated agents.

Paclitaxel (PTX) suppresses the polymerization dynamic of microtubules, resulting in mitotic arrests. Resistance to PTX can be mediated by general mechanisms (such as overexpression of the efflux drug proteins) or is associated with molecular mechanisms of the mitotic process, including tubulin isoforms ratio (TUBB3 overexpression), microtubule-associated proteins (Tau, MAP4) or spindle assembly checkpoint protein (Mad2, BubR1, Aurora A) functionality^[12,13]^. Complex analysis of these molecules may be helpful to predict the effect of PTX. Among the various molecular markers of multidrug resistance or PTX resistance, small regulatory RNAs (microRNAs) appear to be a promising class^[14]^. MicroRNAs or miRNAs are involved in the control of all cellular functions, and some of them directly regulate the expression of proteins mediating the response of BC cells to PTX. For instance, miR-125b confers PTX resistance to BC cells through suppression of pro-apoptotic Bcl-2 antagonist Killer 1 (Bak1) expression^[15]^. miR-451 is involved in the resistance of BC cells to PTX by targeting key regulators of cell survival and apoptotic machine 14-3-3ζ proteins (YWHAZ)^[16]^. miR-152 suppresses endothelial PAS domain-containing protein 1 (EPAS1), thereby enhancing the apoptosis rate and susceptibility of BC cells to PTX^[17]^. miR-155 contributes to PTX resistance of BC cells through tumor protein p53 inducible nuclear protein 1 (TP53INP1)^[18]^. miR-16 sensitizes BC cells to PTX through the suppression of IKBKB expression^[19]^. miR-520h stimulates PTX resistance by targeting the OTUD3-PTEN axis in BC cells^[20]^. Scientific data indicate the involvement of specific miRNA molecules in controlling PTX resistance. However, only a limited number of systemic investigations of the miRNA-mediated response of BC to PTX have been performed so far and the results of these studies are hardly comparable. Uhr *et al.* investigated the expression of 411 miRNAs in the context of sensitivity to 34 drugs (including PTX) in 36 BC cell^[21]^. This large-scale experimental study showed an association between PTX sensitivity and miR-187, miR-106a, and miR-556 expression; however, these results were relevant to *in vitro* cultured cells only and were not evaluated in BC tissues. In contrast, Wu *et al.* investigated the network of transcription factor-miRNA-mRNA in samples of BC tissues (n. 50) before and after treatment with PTX^[22]^. They observed an association between the effect of PTX and the expressional profile shift of several miRNAs (miR-508, miR-4445, miR-3545, miR-1911, miR-584, miR-4782, and miR-219). These exciting computational data require experimental validation.

In our study, we attempted to combine and take advantage of both experimental and observational approaches. First, potential markers of BC cell resistance to PTX were identified by combined analysis of miRNA expression profiles and intrinsic PTX sensitivity of five BC cell lines. Second, the expressions of selected miRNAs were analyzed in tumor tissues of patients with BC and their association with the effect of PTX-based NAT was verified. As the mechanism of PTX action is not related to the HR/HER2 status, patients with different BC subtypes were included in the study. The NAT of all patients was started with PTX, which allowed us to attribute the effect of the first two cycles to PTX sensitivity only. With this approach, we could identify miRNAs that are most likely associated with the phenomenon of inherent resistance of BC cells to PTX.

## METHODS

### BC cell lines

Cell lines were obtained from the Institute of Cytology (Russian Academy of Sciences). MCF7, BT20, MDAMB231, and MDAMB453 are widely used BC tissue-derived cell lines. The HBL-100 line was obtained from primary cultures of cells derived from an early lactation sample of human milk^[23]^; however, following studies revealed the presence of Y chromosome and malignant characteristics of these cells^[24,25]^. Small tandem repeat (STR) profiling was performed as a commercial service (InLab-genetics.ru) to confirm the identity of all used cell cultures [Supplementary Appendix 1-5]. All cell lines were cultured in RPMI 1,640 medium (PanEco, Moscow, RF) supplemented with 10% heat-inactivated fetal bovine serum (Biosera, Nuaille, France) and 1% penicillin-streptomycin (PanEco, Moscow, RF) at standard conditions.

### Immunohistochemistry

Cells were cultivated on 4-chamber slides till they reached sufficient confluence. They were washed with PBS, fixed with 4% paraformaldehyde, permeabilized (for nuclear receptors assay) with 0.1% Triton X-100, blocked with 2% BSA, incubated with rabbit antibodies against ER, PR, or HER2 (Ventana Medical System Inc., Tucson, USA, cat. No. 790-4,325, 790-429, 6,790-4,493) and stained following the protocol specified in the detection kits (Ventana Medical System Inc., Tucson, USA).

### Flow cytometry

Cells in suspension (1 million cells/250 µL) were treated with BD Cytofix/Cytoperm Kit (BD Bioscience, Heidelberg, Germany), washed with PBS, incubated with antibodies against ER, PR, or HER2 (Ventana Medical System Inc., Tucson, USA, cat. No. 790-4,325, 790-429, 6,790-4,493), washed again and stained with secondary anti-rabbit FITC-labeled antibodies (Abcam, Cambridge, UK, cat. No. AB6717). Analysis was performed using a CytoFLEX cytometer (Beckman Coulter, Brea, USA).

### PTX toxicity and cell viability assays

Cells were seeded into 96-well plates and incubated till they reached 50% confluence. PTX (Sigma-Aldrich, St Louis, USA) was dissolved in DMSO to make the stock concentration of 2 mm, and working concentrations (200-1 nM) of the drug in a complete medium were prepared by serial dilution. PTX was applied to the cells at different concentrations by medium replacement. Control cells were treated with 0.01% DMSO in a complete medium, which corresponded to the amount of DMSO in PTX-treated cells at a maximum concentration (200 nM). Cells were incubated for 72 h and cell viability was assayed with the WST kit (Abcam, Cambridge, UK) following the manufacturer’s protocol. The absorbance was measured at 450 nm using a multi-plate reader, Varioscan LUX (Thermo Fisher Scientific, Wolfteam, USA). Each measurement was done in triplicate and the results reflected the average value. The cell viability was expressed as a percentage of viable untreated cells (Mean ODsample/Mean ODcontrol × 100).

### Patients

The study design was approved by the Ethics Committee of Petrov’s NMRC of oncology 04.02.2021. All patients involved in the study signed the informed consent form. The diagnostic evaluation included whole breast ultrasound (US) investigation of the tumor and core biopsy; the NAT was started with PTX. The effect of therapy was evaluated after the first two cycles. The majority of patients (n. 18) included in the study received PTX monotherapy. In these cases, the expected effect was achieved, which allowed the operation to be performed. In some patients, the clinical effect of PTX was worse and after 6 (n. 4) or 12 (n. 9) cycles, PTX was replaced by a combination of doxorubicin and cyclophosphamide (AC). Radiation therapy before surgery was not applied in any of the patients. All clinical data obtained were depersonalized before analysis.

### RNA isolation

Total RNA was extracted from the cell pellets containing one million cells with miRNeasy Mini kit (Qiagen, Hilden, Germany) following the manufacturer’s protocol to analyze miRNA from BC cells. To analyze miRNA from BC tissues, material obtained from diagnostic biopsy was used. After morphological and IHC confirmation of the diagnosis, formalin-fixed, paraffin-emdedded (FFPE) tissue samples were sectioned, deparaffinized by incubation with mineral oil and ethanol, and treated with proteinase K (Sileks Ltd., Moscow, RF). The digested samples were centrifuged at 10,000 g for 10 min and the supernatant was mixed with lysis buffer (guanidine thiocyanate, 2 m; sodium acetate pH 4 and 0.6 m; octanic acid, 0.5%, and 2% 2-mercaptoethanol), incubated for 3 min and centrifuged at 10,000 g for 3 min. The supernatant was mixed with 400 μL of 95% ethanol and transferred into a silica spin column (BioSilica Ltd., Moscow, RF). The columns were washed twice with washing buffer and centrifuged to remove excess liquid. RNA was eluted with 120 µL RNA elution solution (EDTA pH 9,510 mm). The RNA quality and concentration were assayed with a Qubit fluorimeter (Thermo Fisher Scientific, Wolfteam, USA).

### miRNA analysis

To profile miRNA in the culture of BC cells, the cDNA library was prepared using the Illumina TruSeq Small RNA library preparation Kit following the manufacturer’s instructions. The libraries were sequenced on the Illumina HiSeq 3,000 platform, and sequencing reads were aligned with the STAR^[26]^ and normalized using DESeq2^[27]^. To assay the expression of selected miRNAs in BC tissue samples, reverse transcription was performed with two-tailed primer^[28]^ followed by PCR using ALMIR-miRNA Assay Kits and protocols (Algimed Techno, Minsk, Belarus) and Real-Time CFX96 Touch instrument (Bio-Rad, Hercules, USA).

### Statistical treatment and software

Statistical analyses were performed and images were drawn using Microsoft Excel 2016 (Microsoft Corporation, USA), CytExpert Software 3 (Beckman Coulter, Inc., USA), Quest Graph™ IC50 Calculator (AAT Bioquest, Inc., USA), OriginPro 9.1 software (OriginLab Corporation, USA), GraphPad Prism software (GraphPad Software Inc., USA). To evaluate the statistical significance of the correlation between two parameters, the Pearson correlation coefficient (Pearson’s r) was estimated. To evaluate the statistical significance of the difference between the two groups, the non-parametric Mann-Whitney test was applied.

## RESULTS

### Study design

**Figure 1:**
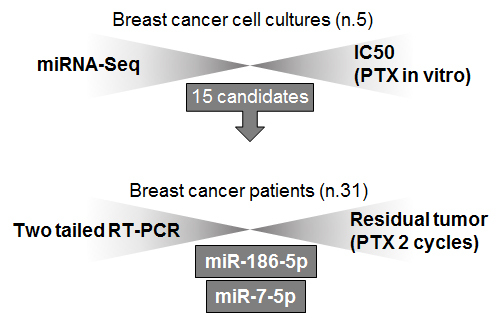
Study design.

To select miRNAs potentially associated with the response of BC cells to PTX, five cell lines were explicitly characterized for morphology, HR/HER2 status, PTX sensitivity, and miRNA profiling. This step allowed us to select 15 candidate molecules whose expression level was correlated with PTX sensitivity. The expression of these miRNAs was then assayed by RT-PCR in BC tissues from patients (n. 31) with different effects of PTX-based therapy [Figure 1].

### BC cell line characteristics

To model the diversity of BC, we used five different BC cell cultures (MCF7, BT20, MDAMB231, MDAMB453, and HBL100). All cell cultures included in the study were established and characterized explicitly^[29-33]^. Given that many passages can alter the characteristics of the cells, we evaluated their STR profiles and confirmed their identity [Supplementary Appendix 1-5]. The cells exhibited different morphologies [Supplementary Figure 1], thus reflecting the natural diversity of BC. Next, we analyzed the expression of steroid hormone receptors (estrogen receptor, ERα; progesterone receptor, PG) and receptor of epidermal growth factor (HER2). Expression of ER and PR evaluated by IHC using antibodies routinely used for BC tissue staining was almost non-detectable in all cell lines (not shown). Flow cytometry is an additional approach to validate ER, PR and HER2 marker expression^[34]^. Herein, flow cytometry was found to be more sensitive than IHC. Results are presented in Table 1.

**Table 1:** Expression of ER, PR and HER2 in BC cell lines assayed by flow cytometry

**REC\CELLS**	**MD A321**	**B T 20**	**MC F7**	**MD A435**	**H B L100**
**ER**	1.78%	1.25%	4.64%	2.14%	1.39%
**PR**	1.60%	1.37%	3.52%	6.98%	1.71%
**HER2**	0.55%	2.41%	1.81%	76.25%	1.33%

Results are presented as a percentage of positive cells estimated as (positive cells · 100)/all assayed cells using CytEXPERT software. The difference in the expression of each marker within the five cell cultures is additionally demonstrated by color, while the maximum expression level is indicated by a completely shaded area, and the lower expression level is indicated by a proportionally smaller colored area.

**Figure 2:**
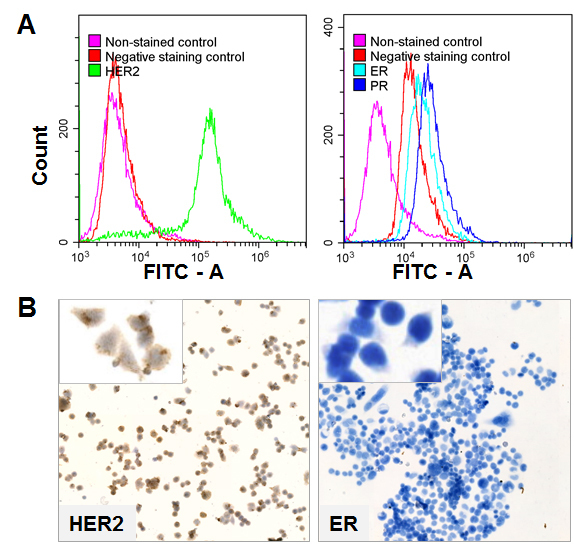
Expression of HER2, ER, and PR in MDAMB453 cell culture evaluated by (A)Flow cytometry; and (B) immunohistochemistry. Images are shown at 10x and 40x magnification.

We confirmed the ER- and PR-positive status of MCF7 cell line using flow cytometry. Moreover, other cell lines also showed ER and PR staining, but the signals were weaker than that of MCF7. This unexpected result was obtained with MDAMB453 cells, which were ER- and PR-positive as shown in Figure 2A (color legend are the same as in Figure 2) and confirmed by IHC [Figure 2B].

These observations contradict previously published data^[32,33]^ and may reflect the modification of cell phenotype or show the high sensitivity of flow cytometry.

### PTX sensitivity of BC cells *in vitro*

**Figure 3:**
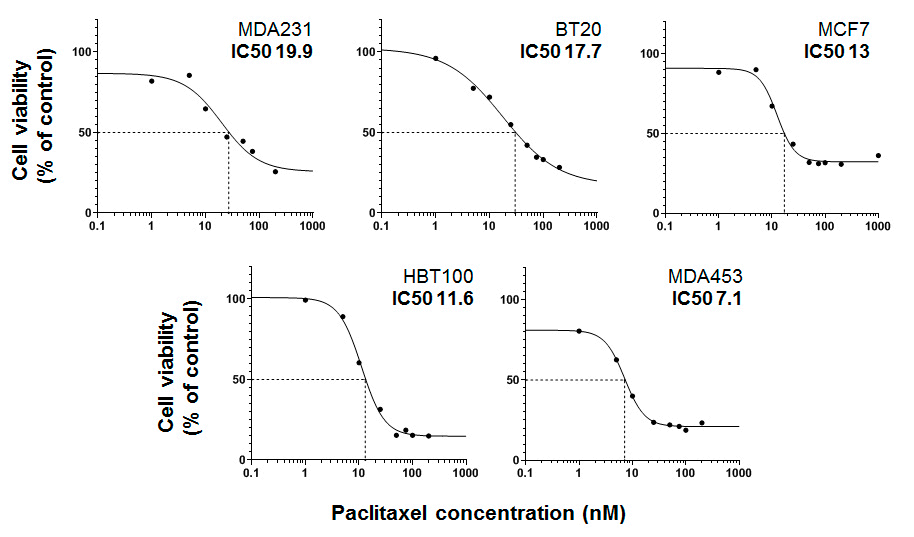
Sensitivity of different BC cell cultures to PTX *in vitro* evaluated by dose-dependent cell viability assay. PTX: Paclitaxel.

All five cell lines were treated with PTX with different concentrations for 72 h and WST test was used to evaluate cell viability. IC50 values were calculated using the Quest Graph™ IC50 Calculator [Figure 3].

The PTX IC50 (IC50-PTX) values in tested cell lines varied considerably, ranging from 7.1 nM to 19.9 nM. MDAMB231 cells (derived from basal-like BC) with specific stellate morphology and low expression levels of ER, PR, and HER2 were relatively resistant to PTX. The BT20 cell line also established from basal-like tumors with similar morphological properties and receptor status was resistant, but slightly lesser (IC50-PTX: 17.7 nM). Maximal sensitivity to PTX was observed in MDAMB453 cells derived from luminal BC with detectable levels of nuclear sex hormones receptors and high expression of the surface receptor, HER2. Both the MCF7 [ER (+)/PR (+)] derived from luminal BC and normal mammary epithelium cell line HBL-100 showed intermediate PTX sensitivity. Thus, cell lines derived from basal-like tumors with low levels of growth factor receptors were relatively resistant to PTX, whereas cell cultures derived from luminal BC or normal mammary epithelium with higher levels of growth factor receptors were more sensitive to PTX.

### miRNA expression profiling in BC cell lines

**Figure 4:**
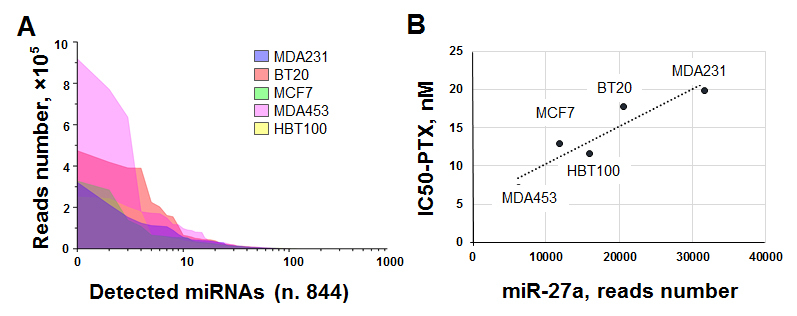
Sequencing of miRNAs in BC cell lines. (A) Overall frequency distribution of detected miRNAs schematically presented using OriginPro 9.1 software; and (B) representative example of the correlation between miRNA-27a expression level and IC50-PTX presented graphically using Microsoft Excel 2016. BC: Breast cancer; PTX: paclitaxel.

The main goal of our *in vitro* experiments was to evaluate the possible link between miRNA expression patterns and inherent sensitivity to PTX. To assay a broad profile of cellular miRNAs, we performed sequencing. After bioinformatics analysis of miRNA sequencing data, 844 miRNAs were identified at least in one of five cell lines [Supplementary Table 1], with 705 to 750 different miRNAs obtained in each cell line. The total number of reads for each miRNA in each cell type varied from zero to 900,000, indicating a broad range of miRNA expression. Comparative representation of different miRNAs in analyzed samples is graphically shown in Figure 4A using the log scale on X-axis.

Thus, only 250 miRNAs in BT20 cells were quantified by more than 100 reads-292 miRNAs-in HBL100 cell culture, 265 miRNAs in MCF7, 247 miRNAs in MDAMB231, and 245 miRNAs in MBAMD453. Only 145 miRNA molecules reached 100 reads in all five cell lines tested, suggesting that relatively small numbers of miRNAs are represented typically and abundantly in studied cells. These miRNAs play a role in controlling basal cellular functions. Other molecules are less abundant and more variable across cell lines; these miRNAs can mediate temporal expressional alterations and control specific functions.

Next, we estimated the linear correlation between two sets of data (IC50-PTX *vs.* normalized read count for each miRNA) in five cell lines using the Pearson correlation coefficient (Pearson’s r). This coefficient exceeded the value of 0.8 for 48 miRNAs and exceeded 0.9 for 18 miRNAs including cases in both direct and inverse relationships [Supplementary Table 1]. An example of such correlation for miR-27a is illustrated in Figure 4B. We concluded that these molecules might be involved in the response of cells to PTX. In this case, their expression level and functionality might predict the cytostatic effect of the drug. To narrow the list and minimize the number of random matches, relevant scientific data analysis was performed using the PubMed library with the keyword combination of “breast cancer AND paclitaxel AND miR-XXX.” Fifteen miRNAs, listed in Table 2, along with the value of Pearson’s r and relevant references, were selected for expressional analysis in BC tissue samples.

**Table 2:** List of miRNAs selected for expressional analysis in BC tissue samples

**miRNA**	**miRNA expression (reads number) in specific cell lines**					Pearson’s r*	**Ref.**
	**MDA231**	**BT20**	**MCF7**	**HBL100**	**MDA453**		
125b-5p	1391	2045	3818	5615	12236	-0.93	[15,35]
145-5p	69888	22409	4489	9	69	0.83	[35]
16-5p	5797	6116	2798	160	115	0.93	[19]
186-5p	11	74	202	625	1161	-0.93	[36]
191-5p	10191	5735	2128	250	5	0.93	[37]
200c-3p	182	56	528	387	1066	-0.91	[38,39]
221-3p	60509	40509	813	10509	222	0.91	[40]
24-3p	22916	19121	1150	11916	1127	0.87	[41]
27a-3p	31571	20618	11817	15958	6216	0.93	[42]
29-3p	10	11	5	5	1	0.97	[42]
30a-5p	6182	5129	290	2144	65	0.91	[43]
34a-5p	8112	7971	1328	3246	216	0.93	[44]
451a-5p	51371	47166	23124	1321	4982	0.92	[16]
7-5p	135	199	370	586	692	-0.97	[45]
93-5p	6760	18488	18175	28622	80353	-0.87	[46,47]

*Pearson coefficient for linear correlation between two sets of data (IC50-PTX *vs.* normalized read count for each miRNA) in five cell lines. PTX: Paclitaxel.

### Expressional analysis of candidate miRNAs in BC tissue samples

BC tissue samples were obtained through diagnostic core biopsy and used for routine histological and IHC evaluation. The patients assigned to the PTX-based NAT group were included in the study and samples of their BC tissue were used for expressional assessment of the selected miRNAs. Characteristics of patients and tumors are presented in Table 3.

**Table 3:** Characteristics of BC patients (n. 31) included in the study

**Characteristics**		**Groups**	**N (total number 31)**	**%**
Clinical status	Age	< 50	18	58
		≥ 50	13	42
	Clinical T	T 1-2	25	81
		T 3-4	6	19
	Clinical N	N 0	6	19
		N +	25	81
Histology (trepan biopsy)	Grad	G 1-2	22	71
		G 3-4	9	29
	Ki67	< 20	8	26
		≥ 20	23	74
	ER	Pos	29	94
		Neg	2	6
	PR	Pos	26	84
		Neg	5	16
	HER2	Pos	3	10
		Neg	28	90
Neo-adjuvant therapy^*^	6-12 PTX		18	58
	6 PTX - AC		4	13
	12 PTX - AC		9	29
PTX effect evaluation (US)	Residual tumor size after 2 cycles of PTX (% from initial volume)	< 50	14	45
		≥ 50	17	55
Completed scheme therapy effect evaluation (histology)	Miller and Payne system	Grade 1-2	8	26
		Grade 3-4	20	65
		Grade 5	3	10
	Residual cancer border class	I	1	3
		II	16	52
		III	11	35
		pCR	3	10

^*^The schemes of neoadjuvant chemotherapy are shown by number of PTX cycles applied as monotherapy followed by combination of doxorubicin and cyclophosphamide (AC). PTX: Paclitaxel.

Tumor size (before and after two cycles of PTX) was measured by whole breast ultrasound (US) in three dimensions (width, W; length, L, and height, H) and tumor volume was approximated assuming a rounded shape using the following equation: V = 4/3 · π · 1/2W · 1/2L · 1/2H. Neoadjuvant chemotherapy was started with an intravenous infusion of PTX at a dose of 80 mg/m^2^ weekly as recommended by the national standard^[48]^. The primary effect of therapy was evaluated after two cycles of PTX. Residual tumor volume after two cycles of PTX-based therapy (residual tumor size, RTS) was expressed as a percentage of its initial volume using the following equation: RTS = (100 × V2)/V1. All patients underwent dynamic observation and received optimal therapeutic regimens based on their complex parameters [Table 3]. After NAT was completed, all patients underwent surgery (mastectomy) and the ultimate result of therapy was evaluated histologically [Table 3].

**Figure 5:**
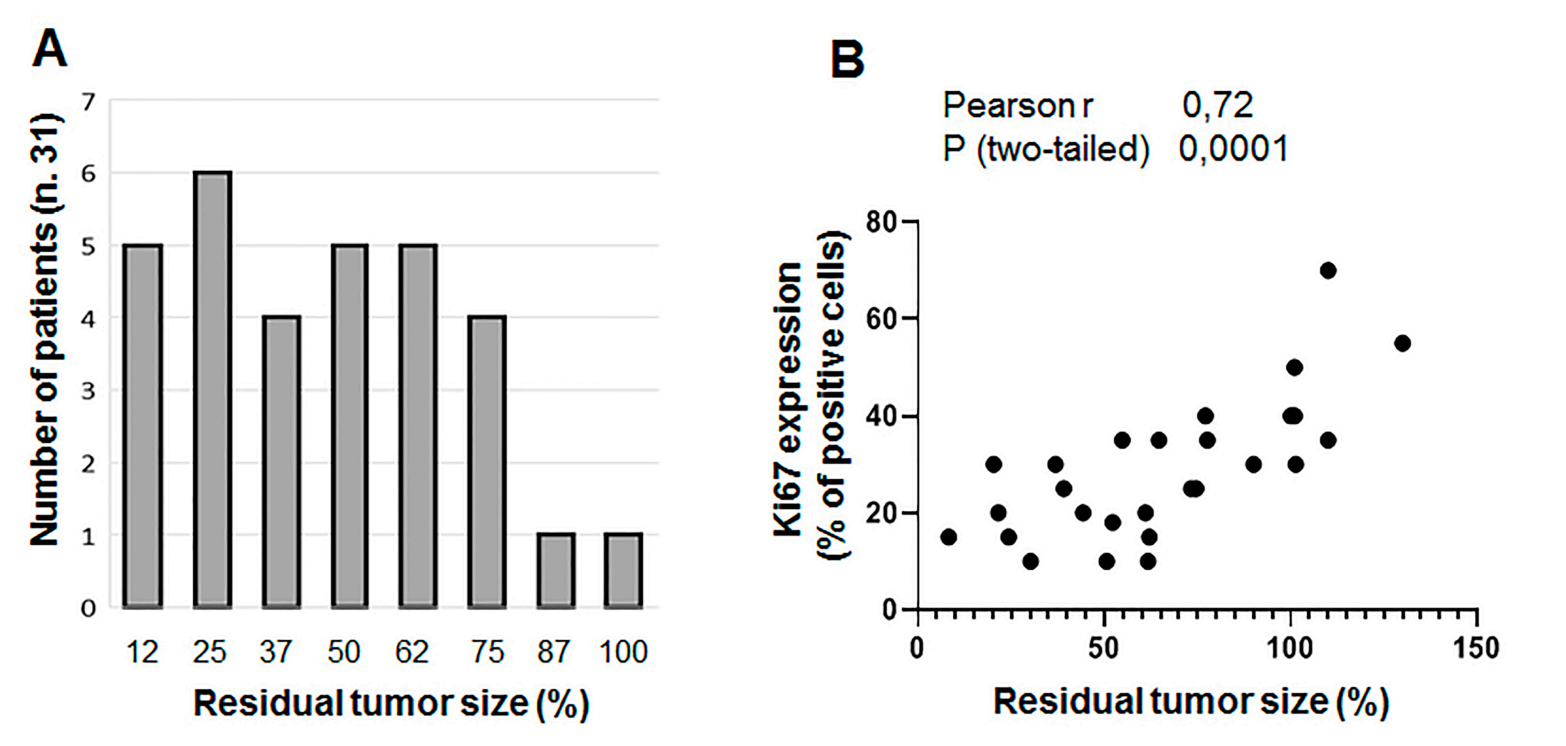
US-based evaluation of PTX-based therapeutic effect. (A) Distribution of patients according to size of residual tumor after two cycles of PTX; and (B) correlation between residual tumor size and Ki-67 expression in specimens from diagnostic biopsy. Both graphs were plotted using GraphPad Prism software. PTX: Paclitaxel.

RTS after two cycles of PTX was a parameter reflecting the effect of a single drug. Interestingly, this parameter had an almost normal (Gaussian) distribution among the analyzed group of patients, reflecting the variability of intrinsic sensitivity of BC to PTX [Figure 5A].

Moreover, residual tumor size expressed as a percentage of initial tumor size directly correlated with Ki67 expression [Figure 5B]. Interestingly, we did not find any correlation between the results of histological evaluation of the completed scheme of NAT and Ki67 expression. This observation may indicate the cumulative nature of the action of PTX when an actively proliferating tumor requires long-term exposure to the drug, while a slowly proliferating tumor is suppressed faster. If this explanation is reasonable, it may take more than two weeks for PTX to exhibit a clinical effect.

**Figure 6:**
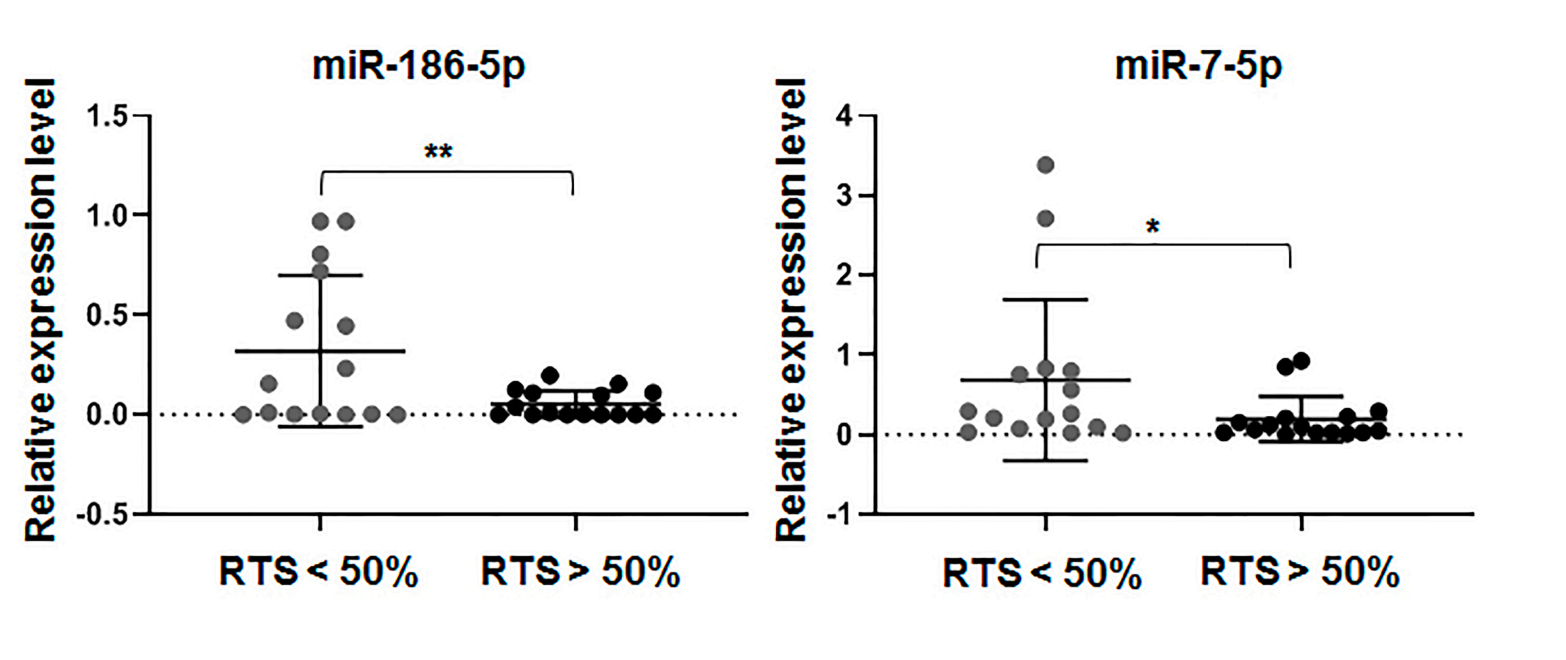
Expression of miRNA (miR-186-5p and miR-7-5p) in BC tissues of patients (n. 31) distributed into two groups with the differential effect of PTX-based therapy evaluated as the relative RTS. Statistical significance was estimated by the Mann-Whitney test (^*^P < 0.05; ^**^P < 0.005). Graphs were plotted and statistical analysis was performed using GraphPad Prism software. BC: Breast cancer; PTX: paclitaxel; RTS: size of tumor residual.

Expressions of 15 selected microRNA were assayed in all 31 samples of BC tissues obtained by core biopsy before the start of NAT. Results were normalized to average Ct (average of 465 individual values, 31 × 15). Subsequently, we attempted to identify the correlation between the expression of each selected miRNA and the known characteristics of the tumor. Patients were distributed into two groups based on tumor residual value after two cycles of PTX, i.e., more (n. 15) and less than 50% (n. 16). Expressions of miR-185 and miR-7 were higher in the tumor group more sensitive to PTX [Figure 6].

**Figure 7:**
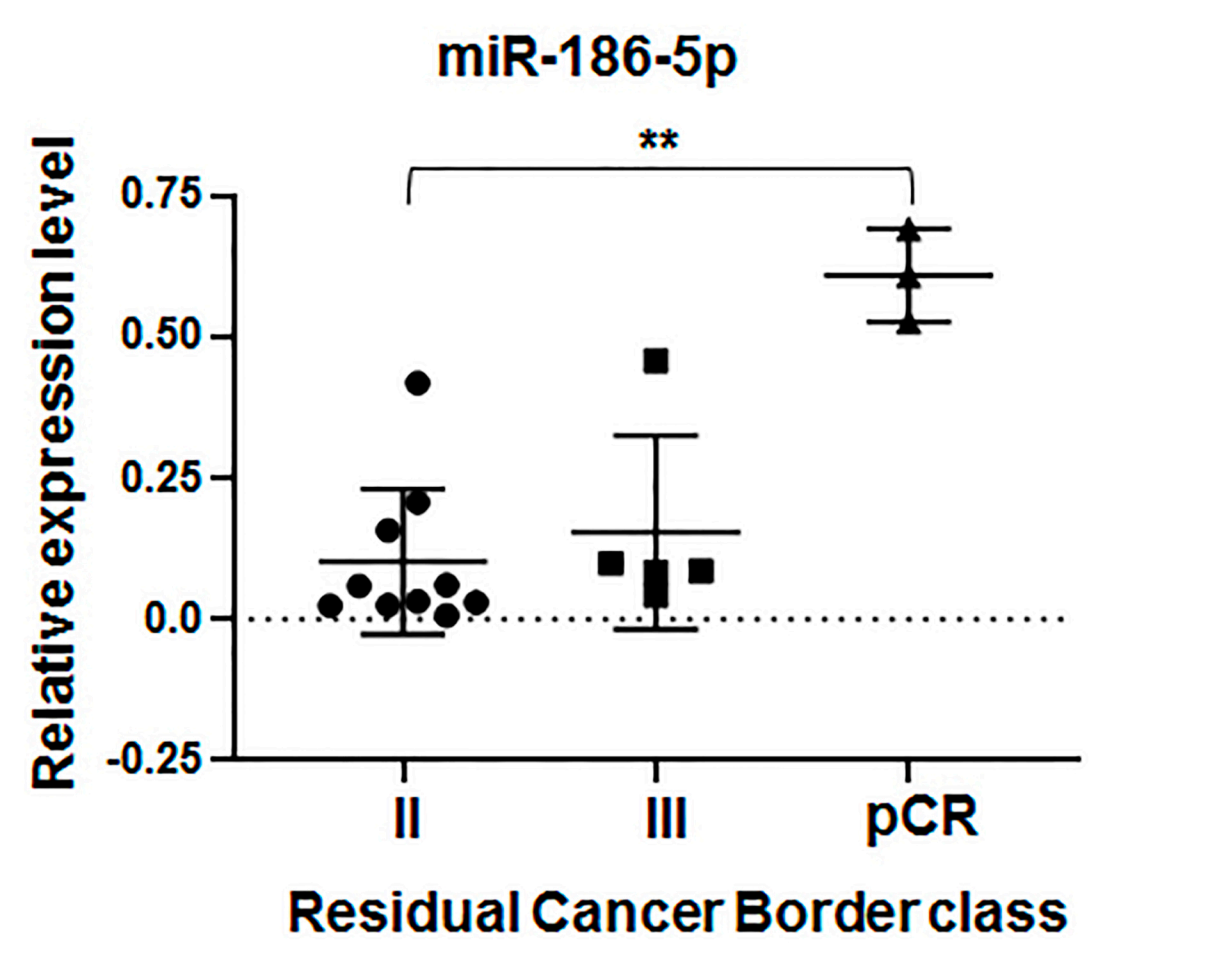
Expression of miR-186-5p in BC tissues of patients (n. 18) distributed into three groups with differential effect of complete scheme of PTX-based therapy evaluated after surgery using Residual Cancer Border Classification. Statistical significance was estimated by the Kruskal-Wallis test (^**^P  < 0,005). Graphs were plotted and statistical analysis was performed using GraphPad Prism software. PTX: Paclitaxel.

We did not find any correlation between the expression of other miRNAs tested and the effect of PTX. Finally, we did not find any correlation between candidate miRNA expression and results of post-surgery histological evaluation of NAT’s effect using either the Miller&Payn system or Residual Cancer Border classification. When the analysis was applied to 18 patients treated only with PTX in the monotherapy regime, a statistically significant difference in miR-186-5p expression level was observed between groups of patients with different Residual Cancer Border statuses [Figure 7].

## DISCUSSION

Several comprehensive genome-wide studies revealed alterations of miRNA profile in BC due to PTX treatment. The list of miRNAs affected by taxane includes molecules implicated in intrinsic resistance^[21,22]^. MiRNAs apparently involved in acquired taxane resistance were identified by comparative analysis of miRNA profiles of docetaxel-resistant BC cell lines and docetaxel-sensitive parental cells^[49]^. However, the results of different genome-wide investigations show negligible overlaps, highlighting the necessity of their confirmation by combining *in vivo* and *in vitro* approaches. Other studies combine BC tissue assessment and *in vitro* experiments, which usually focus on the molecular function of certain miRNAs only (12-17) and aim to identify new approaches to overcome taxane resistance.

This study aimed to identify the most reliable prognostic miRNA markers. As an example of a similar approach, the assay of global miRNA expression in BC tissue of patients with and without recurrence after endocrine therapy followed by validation of selected miRNAs identified miR-30s, -30b, -182, and -200c as independent predictors of clinical benefit from endocrine therapy^[50]^. We attempted to identify miRNA predictors of BC cell sensitivity to PTX, and an advantage of our study was a combination of experimental and observational approaches. First, we selected miRNAs whose expression levels were correlated with PTX sensitivity of BC cells *in vitro*. Second, we explored the correlation between the expression of these miRNAs in BC tissues and PTX-based NAT effect. We believe that this strategy helped us to increase confidence in the obtained results and identify miRNAs truly involved in the response of BC cells to PTX. As the mechanism of action of PTX is not associated directly with HR and/or HER2 status^[51,52]^, in our study, we analyzed BC tissue specimens from patients with different subtypes of BC, mostly HR+ /HER2-, and we did not attempt to evaluate any correlation between PTX effect and BC subtypes. The design of our study allowed the evaluation of the link between high levels of miR-186-5p and miR-7-5p expression and the effect of two weekly cycles of PTX. The effect of the complete scheme of NAT was associated with high expression of miR-186-5p in a group of patients treated with PTX only. In patients assigned to the neoadjuvant polychemotherapy (PTX-AC) group, no correlation between tested miRNA expression and residual cancer border classes was found. This might highlight the specific association between miR-186-5p expression and PTX sensitivity.

The role of miRNA-186 in cancer is well-studied. Recent reviews have described the dual properties implicated in carcinogenesis and the involvement in various cellular processes^[53,54]^. In the context of BC, miR-186 was reported as an onco-miR that mediates highly aggressive and metastatic phenotype of tumor^[55]^. However, miR-186 sensitized non-small lung cancer (*in vitro* and *in vivo*) to PTX by modulating MAPK activity^[56]^. After computational prediction of the miR-186 binding site in 3’UTR of ABCB1, the mechanism of miR-186-mediated sensitivity to PTX was evaluated in *in vitro* models of ovarian cancer^[57]^. As demonstrated here, the expression of miR-186 was higher in BC cell lines sensitive to PTX and in samples of BC effectively treated by PTX. Therefore, miR-186 may sensitize BC cells to PTX through mechanisms described previously for other cancers.

The link between miR-7 expression and PTX sensitivity observed in our study is consistent with results published by other groups. Interestingly, the sensitization to PTX by miR-7 was reported in the same cancer types as miR-186, including non-small cell lung and ovarian cancer^[58,59]^. The mechanisms of miR-7 action were non-related to microtubular apparatus and had universal character (regulation of EGFR/ERK signaling). miR-7 was significantly overexpressed (fold change > 3) in response to PTX treatment in a cell line (FaDu) derived from hypopharyngeal tumor^[60]^, reflecting the implication of this molecule in the adaptive reaction of cells to the drug. As reported recently^[45]^, in BC cells, miR-7 reversed resistance to PTX by targeting both the multidrug resistance-associated protein 1 (MRP1) and anti-apoptotic B cell lymphoma 2 (BCL2).

In conclusion, presented results showed that miR-185 and miR-7 expressions are higher in PTX-sensitive BC cells than in resistant cells. It was demonstrated for the first time that the overexpression of these molecules in BC tissues is associated with sensitivity to PTX, whereas their downregulation may reflect resistance to PTX applied as monotherapy. The innovative potential of the presented results lies in the possibility of developing new approaches for predicting the effect of PTX therapy based on the analysis of miRNAs in biopsy material. Since different miRNAs reflect the intrinsic resistance of BC to different drugs, a miRNA prediction panel can be developed and proposed as an additive approach for the personalized choice of NAT regimen.

## DECLARATIONS

### Copyright

© The Author(s) 2023.
