# Supplementary material for: Resistance of breast cancer cells to paclitaxel is associated with low expressions of miRNA-186 and miRNA-7
Source: Cancer Drug Resist. 2023 Sep 1;6(3):596–610. doi: 10.20517/cdr.2023.19 (PMC10571055; doi:10.20517/cdr.2023.19)
Supplement: Supplementary file 1 [file cdr-6-3-596-SupplementaryMaterials.pdf]

AN EXPRESSION OF MIR-186 AND MIR-7 IS INCREASED IN BREAST CANCER CELLS SENSITIVE TO PACLITAXEL

Vera Apollonova, Daniil Plevako, Alexandr Garanin, Elena Sidina, Lidia Zabegina, Margarita Knyazeva, Viktoria Smirnova, Anna Artemyeva, Petr Krivorotko, Anastasia Malek

Supplementary Figure S1: Morphology of BC cell cultures.

Images were obtained in 40x magnification with MIB-R inverted microscope supplied with camera (LOMO-Microsystems, Russia).

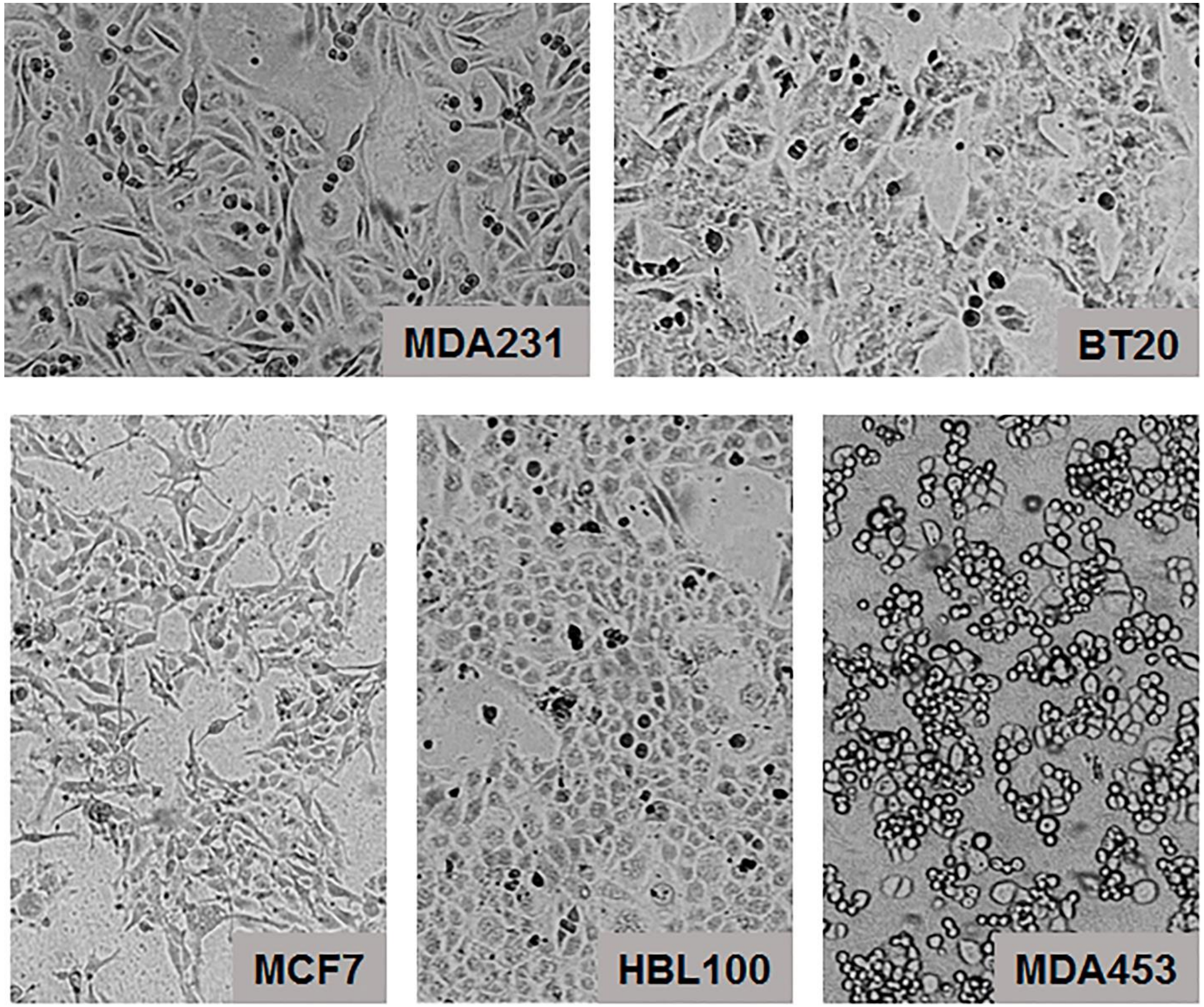

Supplementary Table S1: miR-seq complete results

The numbers reflects reads of single miRNA in individual cell line. Data were normalized with DESeq2 algorithm developed by Simon Anders and colleagues (1) for comparative RNA-seq data using shrinkage estimators for dispersion and fold change.

Pearson's correlation coefficients R reflect a linear (direct or inverse) correlation between the level of expression of a specific microRNA in five cell lines and the sensitivity of these cells to paclitaxel, estimated as a half-maximal concentration of inhibition of cell viability (IC50 value).

miRNA selected for further analysis are indicated by dark background.

MIR	MDAMB231	BT20	MCF7	HBL100	MDAMB453	PEARSON R
HSA-MIR-29-3P	10	11	5	5	1	0,966769171
HSA-MIR-140-5P	7258	5099	979	1104	147	0,941451358
HSA-LET-7C-5P	757	552	74	202	17	0,932548943
HSA-MIR-27A-3P	31571	20618	11817	15958	6216	0,93209688
HSA-MIR-34A-5P	8112	7971	1328	3246	216	0,931714099
HSA-MIR-16-5P	5797	6116	2798	160	115	0,929870235
HSA-MIR-424-5P	13788	9324	4297	1109	1482	0,929355796
HSA-MIR-191-5P	10191	5735	2128	250	5	0,927582446
HSA-MIR-451	51371	47166	23124	1321	4982	0,924372848
HSA-MIR-382-3P	9406	8517	2996	106	872	0,920774064
HSA-MIR-874-5P	213352	225249	62803	20	5771	0,920297412
HSA-MIR-659-3P	42	36	6	2	3	0,915062179
HSA-MIR-30A-5P	6182	5129	290	2144	65	0,912502562
HSA-LET-7G-3P	1401	1615	389	19	16	0,911427034
HSA-MIR-221-3P	60509	40509	813	10509	222	0,910289262
HSA-MIR-7113-5P	48	43	8	6	6	0,908267766
HSA-MIR-5000-5P	14	14	10	6	7	0,90552206

**HSA-MIR-4532**	778	757	281	16	108	0,905296626
**HSA-MIR-6087**	100	56	14	3	4	0,904773302
**HSA-MIR-1910-5P**	172	127	69	6	28	0,904275005
**HSA-MIR-877-5P**	642	714	472	107	92	0,90079754
**HSA-MIR-6514-5P**	86	53	7	3	5	0,892685176
**HSA-MIR-3173-5P**	3084	3813	2116	80	224	0,875747904
**HSA-MIR-218-1-3P**	321584	391159	59576	19	20220	0,875676982
**HSA-MIR-196B-5P**	38349	50908	21240	38	2829	0,870223686
**HSA-MIR-24-3P**	22916	19121	1150	11916	1127	0,867841587
**HSA-MIR-29B-2-5P**	64	93	43	17	10	0,863851052
**HSA-MIR-450A-2-3P**	10237	7450	4893	17	2281	0,859804482
**HSA-MIR-583**	66	25	12	2	2	0,85807349
**HSA-MIR-6877-5P**	18	6	3	4	1	0,841233788
**HSA-MIR-145-5P**	69888	22409	4489	9	69	0,834971294
**HSA-MIR-381-5P**	4436	3933	1759	19	1394	0,827673814
**HSA-MIR-197-3P**	1495	1089	498	759	660	0,821768325
**HSA-MIR-497-5P**	4655	6378	2463	25	1170	0,813189645
**HSA-MIR-4463**	64	19	6	3	2	0,812359841
**HSA-MIR-660-5P**	268	194	151	169	158	0,811036572
**HSA-MIR-6763-5P**	54	14	8	3	1	0,809598233
**HSA-MIR-616-3P**	4978	1375	58	12	2	0,808909454
**HSA-MIR-3922-5P**	112	102	64	19	51	0,808209192
**HSA-MIR-1293**	112801	202910	55479	1209	6637	0,801696756
**HSA-MIR-152-5P**	6343	9766	5755	33	727	0,794396693
**HSA-MIR-18A-3P**	631	1035	658	28	2	0,790231971
**HSA-MIR-620**	10	19	6	2	1	0,789029446
**HSA-MIR-509-3P**	51	20	9	5	10	0,787877841
**HSA-MIR-760**	192	183	1	145	1	0,786679295
**HSA-MIR-6767-5P**	10	17	7	2	2	0,786372303
**HSA-MIR-4762-3P**	18	26	6	4	7	0,78435262
**HSA-MIR-181B-3P**	281	559	133	38	24	0,775592255

**HSA-MIR-196A-3P**	2589	3115	1638	36	1004	0,77454571
**HSA-MIR-6791-3P**	11	8	3	8	3	0,771809514
**HSA-MIR-628-3P**	143	301	78	47	15	0,760904658
**HSA-MIR-655-3P**	174	78	17	91	21	0,760415433
**HSA-MIR-499A-3P**	62	34	25	6	23	0,752984836
**HSA-MIR-4769-3P**	3	5	3	1	1	0,743451791
**HSA-MIR-6733-5P**	23	47	20	6	4	0,743140336
**HSA-MIR-184**	44149	45869	53773	28	59	0,736020995
**HSA-MIR-95-5P**	23	6	13	5	3	0,726632764
**HSA-MIR-3940-5P**	124	14	3	3	1	0,72429127
**HSA-MIR-4786-5P**	665	262	191	19	201	0,709060027
**HSA-MIR-4676-3P**	117	282	57	21	22	0,699810323
**HSA-MIR-665**	1162	2766	1335	1228	2	0,690398152
**HSA-MIR-10A-3P**	21428	1289	7146	240	8	0,689430507
**HSA-MIR-3607-3P**	51	95	68	17	15	0,682649855
**HSA-MIR-4804-5P**	11648	278	16	55	199	0,667751861
**HSA-MIR-6766-5P**	614	110	234	1	99	0,667524514
**HSA-MIR-3928-3P**	1129	43	6	197	2	0,663281604
**HSA-MIR-378F**	42	74	60	5	11	0,660965642
**HSA-MIR-576-5P**	7398	1919	1422	337	1937	0,658685785
**HSA-MIR-3138**	7335	5802	9580	28	9	0,657623317
**HSA-MIR-450B-5P**	1025	51	12	217	14	0,657353589
**HSA-MIR-887-3P**	35	92	12	25	7	0,656327171
**HSA-MIR-378C**	2221	6677	486	1	326	0,647528791
**HSA-MIR-185-3P**	114	213	61	111	60	0,636969684
**HSA-MIR-3157-5P**	42322	110141	54825	21	10495	0,636932052
**HSA-MIR-3189-5P**	974	2405	680	3	399	0,636635299
**HSA-MIR-2116-5P**	336	1227	204	19	8	0,635000662
**HSA-MIR-139-5P**	777	1050	493	166	558	0,620820852
**HSA-MIR-3194-5P**	8396	13212	6845	3	5394	0,60792974
**HSA-MIR-6869-5P**	14	28	2	3	8	0,602317978

**HSA-MIR-4668-5P**	8	6	1	9	1	0,593662273
**HSA-MIR-6827-5P**	3	9	3	1	1	0,585116733
**HSA-MIR-548V**	119	160	124	17	80	0,583905442
**HSA-MIR-615-5P**	2023	1646	1622	16	1165	0,583549214
**HSA-MIR-3157-3P**	4368	2001	1727	6	2102	0,579842363
**HSA-MIR-548A-3P**	147	73	107	22	72	0,571953593
**HSA-MIR-411-3P**	15800	13206	15541	84	8387	0,564968947
**HSA-MIR-2110**	265	619	389	110	163	0,557582548
**HSA-MIR-320C**	114	442	161	3	45	0,555827156
**HSA-MIR-874-3P**	278	299	422	82	97	0,552679151
**HSA-MIR-1185-1-3P**	4915	112	151	210	932	0,550995854
**HSA-MIR-337-3P**	3871	8666	6331	330	1975	0,548634152
**HSA-MIR-103A-2-5P**	23	11	30	9	1	0,538038906
**HSA-MIR-570-3P**	16217	61836	28487	24	6901	0,536345602
**HSA-MIR-4779**	13	3	3	6	4	0,536330578
**HSA-MIR-582-5P**	144	360	192	22	98	0,535550678
**HSA-MIR-6505-3P**	4	11	1	6	2	0,519373702
**HSA-MIR-1226-3P**	367	11	314	49	1	0,517006964
**HSA-MIR-3691-5P**	61	25	80	25	1	0,515833026
**HSA-MIR-1291**	17	84	8	24	4	0,508846624
**HSA-MIR-210-3P**	13387	10691	10032	169	9064	0,50481467
**HSA-MIR-6747-3P**	40021	41582	34898	16	30252	0,481893447
**HSA-MIR-3940-3P**	32	132	91	1	14	0,471659947
**HSA-LET-7A-3P**	416	14	19	206	67	0,470647474
**HSA-MIR-5583-3P**	11	29	10	24	2	0,467226724
**HSA-MIR-4657**	610	774	817	3	413	0,467096152
**HSA-MIR-7845-5P**	5630	21710	19015	9	741	0,461264128
**HSA-MIR-132-5P**	863	591	376	180	638	0,458406716
**HSA-MIR-4443**	952	14394	594	29	491	0,455999897
**HSA-MIR-652-3P**	41	140	14	99	4	0,447082453
**HSA-MIR-6737-3P**	144	43	58	6	76	0,444942616

**HSA-MIR-652-5P**	80	112	50	22	77	0,44339691
**HSA-MIR-3909**	359	17990	17	26	5	0,441361324
**HSA-MIR-10B-3P**	1882	23009	413	153	1874	0,435548951
**HSA-MIR-744-3P**	38	5	19	13	14	0,432709564
**HSA-MIR-7106-3P**	3	11	3	3	3	0,428499968
**HSA-MIR-4516**	3	6	6	4	2	0,427328076
**HSA-MIR-148A-5P**	21	11682	9	50	8	0,426721579
**HSA-MIR-1537-3P**	9289	13403	9717	16	8501	0,415097779
**HSA-MIR-494-5P**	17000	65665	39748	25	15671	0,414934883
**HSA-MIR-323A-5P**	294	3269	1078	21	358	0,408859817
**HSA-MIR-6783-3P**	360	453	303	6	331	0,408251963
**HSA-MIR-4710**	24	3	17	11	8	0,400041842
**HSA-MIR-6863**	1	2	1	2	1	0,394759669
**HSA-MIR-1910-3P**	347	141	73	6	222	0,392966155
**HSA-MIR-6839-3P**	593	5880	4340	2	101	0,392645112
**HSA-MIR-597-3P**	11	59	18	6	15	0,389296655
**HSA-MIR-4714-3P**	8	11	17	2	4	0,385893939
**HSA-MIR-542-5P**	534	2170	689	121	688	0,384414074
**HSA-MIR-616-5P**	8	3	1	9	2	0,377464826
**HSA-MIR-548W**	3140	2563	1501	6	2665	0,371213268
**HSA-MIR-433-3P**	148	51	25	88	79	0,361674795
**HSA-MIR-6848-5P**	7	2	3	4	3	0,358285714
**HSA-MIR-100-3P**	2252	2471	2018	329	2119	0,358268335
**HSA-MIR-193B-5P**	66	102	30	137	16	0,348911984
**HSA-MIR-495-5P**	56	402	317	26	46	0,348353891
**HSA-MIR-362-3P**	4	19	19	7	1	0,347963287
**HSA-MIR-574-3P**	909	2937	964	1835	849	0,336007491
**HSA-MIR-1306-5P**	40	101	47	92	23	0,325414757
**HSA-MIR-6775-5P**	34	2	22	1	15	0,314347131
**HSA-MIR-125A-5P**	110138	389974	108207	18665	172299	0,305940729
**HSA-MIR-3074-3P**	16	2940	940	17	498	0,293643556

**HSA-MIR-196A-5P**	41842	49640	24210	22603	44278	0,289468567
**HSA-MIR-6724-5P**	31	19	46	35	8	0,287194599
**HSA-MIR-4690-5P**	6	2	2	3	3	0,282682088
**HSA-MIR-193A-5P**	71	47	31	84	39	0,273705024
**HSA-MIR-7854-3P**	8	2	8	1	4	0,27314236
**HSA-MIR-346**	41	472	181	1	132	0,269590872
**HSA-MIR-30B-3P**	241	44718	16702	17	8680	0,269193513
**HSA-MIR-3928-5P**	51	101	44	2	68	0,263748693
**HSA-MIR-6790-3P**	45	9	65	1	16	0,261894556
**HSA-MIR-7106-5P**	1	6	1	4	2	0,261294961
**HSA-MIR-1193**	8	2	8	9	1	0,255864659
**HSA-MIR-6720-3P**	3401	4471	3230	6	3834	0,254298445
**HSA-MIR-1184**	8	8	2	10	6	0,250638458
**HSA-MIR-5096**	25	19	10	48	4	0,229920581
**HSA-MIR-5697**	97	166	303	17	63	0,219070168
**HSA-MIR-6507-5P**	6	8	1	0	7	0,211892416
**HSA-MIR-4275**	96	1798	2216	0	148	0,211442099
**HSA-MIR-29B-1-5P**	339	211	186	362	228	0,211390421
**HSA-MIR-1238-5P**	21	45	42	0	28	0,20903945
**HSA-MIR-615-3P**	62	279	150	287	31	0,194834236
**HSA-MIR-17-3P**	8	534	591	228	1	0,189006796
**HSA-MIR-6504-3P**	17	5	21	5	9	0,184799522
**HSA-MIR-3167**	48	99	54	6	72	0,176282182
**HSA-MIR-1296-3P**	3	16	8	13	3	0,170785366
**HSA-MIR-4677-5P**	34	967	1610	103	2	0,1560639
**HSA-MIR-142-3P**	2395	12380	1361	17260	2	0,144545662
**HSA-MIR-548N**	21	25	17	6	26	0,142176192
**HSA-MIR-154-3P**	64	81	36	124	45	0,114664717
**HSA-MIR-331-3P**	32	2093	4090	217	19	0,111784103
**HSA-MIR-496**	3	40	20	25	10	0,111387688
**HSA-MIR-3653-5P**	2747	19580	5268	118	10399	0,108660791

**HSA-MIR-6720-5P**	4	301	125	17	120	0,096450623
**HSA-MIR-412-3P**	39628	239	105	6	29841	0,095768722
**HSA-MIR-6799-3P**	30	48	19	6	45	0,093142711
**HSA-MIR-2277-3P**	734	711	1424	6	734	0,090918583
**HSA-MIR-6809-3P**	7	90	228	2	2	0,086426573
**HSA-MIR-141-3P**	2130	3407	13827	212	541	0,084484284
**HSA-MIR-3140-3P**	1645	4814	8129	26	2387	0,07999476
**HSA-MIR-3161**	25	3	10	8	18	0,076094839
**HSA-MIR-3133**	4	22	16	1	12	0,074208935
**HSA-LET-7E-3P**	27	2	4	35	10	0,055718909
**HSA-MIR-6768-3P**	35	57	315	2	1	0,055367213
**HSA-MIR-643**	395	1064	2791	17	423	0,050437416
**HSA-MIR-151A-3P**	124592	155480	66312	24234	179140	0,020054593
**HSA-MIR-92A-3P**	1245	4046	1666	9247	3	0,013272873
**HSA-MIR-4754**	6	3	4	2	5	0,009497215
**HSA-MIR-6847-5P**	13	84	229	1	27	0,008329252
**HSA-MIR-381-3P**	3597	2484	8080	10626	48	0,00061257
**HSA-MIR-4677-3P**	7	81	2	152	2	-0,001593029
**HSA-MIR-4766-5P**	470	8	495	6	359	-0,003794459
**HSA-MIR-27B-5P**	21	40	41	61	17	-0,0107112
**HSA-MIR-199A-5P**	1001	1280	3497	6	1242	-0,02057426
**HSA-MIR-30C-1-3P**	28	28	14	98	10	-0,031158497
**HSA-MIR-2114-5P**	3	2	4	2	2	-0,033240667
**HSA-MIR-6856-5P**	11	209	728	1	66	-0,034353134
**HSA-MIR-2355-3P**	42	37	83	80	25	-0,042961871
**HSA-MIR-6873-3P**	80	240	232	23	184	-0,043706868
**HSA-MIR-191-3P**	2077	3875	30235	214	1720	-0,044060335
**HSA-MIR-491-5P**	695	39319	327746	184	123	-0,046424367
**HSA-MIR-9-3P**	55	20	30	173	11	-0,057537728
**HSA-MIR-6837-3P**	1	37	143	1	14	-0,058846341
**HSA-MIR-548AQ-5P**	16	2	4	23	8	-0,058916241

**HSA-MIR-431-3P**	3	56	68	102	1	-0,065773999
**HSA-MIR-429**	8	2	4	28	2	-0,092251633
**HSA-MIR-548L**	2222	707	12	6	2741	-0,095413888
**HSA-MIR-421**	55	2	3	217	5	-0,09724271
**HSA-MIR-574-5P**	2506	1587	1084	11951	441	-0,09730254
**HSA-MIR-188-5P**	11	9	5341	42	3	-0,097908886
**HSA-MIR-3158-3P**	4	3	6	17	1	-0,103766501
**HSA-MIR-23B-5P**	3	5	951	35	1	-0,104367665
**HSA-MIR-23A-5P**	239	2	1	46	238	-0,108772524
**HSA-MIR-1304-3P**	35	70	36	330	2	-0,1097615
**HSA-MIR-296-5P**	28	3	1	139	1	-0,112779952
**HSA-MIR-501-3P**	10	3	6	19	6	-0,127750524
**HSA-MIR-4484**	41	14	6	253	3	-0,13303599
**HSA-MIR-3200-3P**	14	12	18	82	3	-0,136676904
**HSA-MIR-671-3P**	1821	17	6	211	2008	-0,148167265
**HSA-MIR-219B-3P**	210	220	534	6	327	-0,149331181
**HSA-MIR-28-5P**	35	3	5	246	1	-0,154891352
**HSA-MIR-380-5P**	17902	12554	11677	163	25891	-0,155012144
**HSA-MIR-4672**	7	2	3	1	8	-0,160916899
**HSA-MIR-26A-5P**	1375	2732	482	19277	304	-0,162051508
**HSA-MIR-4440**	171	19	22	7	206	-0,17002135
**HSA-MIR-330-3P**	18	9	5	72	11	-0,17028464
**HSA-MIR-548AZ-5P**	4735	3835	5575	7	7064	-0,175315542
**HSA-MIR-15B-3P**	114	228	62	587	141	-0,176461656
**HSA-MIR-1246**	102	37	43	774	28	-0,183090308
**HSA-MIR-4457**	38	2	58	2	40	-0,183463921
**HSA-MIR-369-3P**	35	71	13	792	4	-0,190478993
**HSA-MIR-361-3P**	112	109	202	1175	22	-0,191492323
**HSA-MIR-6514-3P**	333	490	436	6	656	-0,191583472
**HSA-MIR-4766-3P**	6	3	45	31	2	-0,196034327
**HSA-MIR-320A**	1049	1724	944	11830	722	-0,196700865

**HSA-MIR-128-1-5P**	31	28	11576	37	1493	-0,19694379
**HSA-MIR-450A-1-3P**	23	5	21	29	16	-0,197133217
**HSA-MIR-224-3P**	3	0	2	1	3	-0,198060557
**HSA-MIR-1306-3P**	62	115	235	59	113	-0,198587171
**HSA-MIR-548G-3P**	7	9	8	4	11	-0,20026385
**HSA-MIR-146B-3P**	0	2	1	9	0	-0,203114161
**HSA-MIR-4524A-3P**	3	0	0	5	2	-0,203365914
**HSA-MIR-1304-5P**	0	2	0	15	0	-0,206558725
**HSA-MIR-556-3P**	0	2	0	15	0	-0,206558725
**HSA-MIR-877-3P**	0	2	1	10	0	-0,206913082
**HSA-MIR-3934-5P**	0	5	1	30	1	-0,207616778
**HSA-MIR-573**	0	0	9	4	0	-0,208801692
**HSA-MIR-365A-5P**	3	2	2	20	2	-0,210911647
**HSA-MIR-589-3P**	3	0	1	35	1	-0,212481594
**HSA-MIR-500B-3P**	8	2	5	2	9	-0,213664067
**HSA-MIR-449C-5P**	0	2	0	18	0	-0,213777874
**HSA-MIR-3613-5P**	90177	563	308	211	117602	-0,21572354
**HSA-MIR-32-5P**	1	65	1	941	0	-0,219423515
**HSA-MIR-340-5P**	0	48	0	701	0	-0,220462859
**HSA-MIR-1-5P**	47	34	14	0	80	-0,224000193
**HSA-MIR-126-5P**	114	40	1	1399	67	-0,224382067
**HSA-MIR-449A**	1	2	292	79	21	-0,225238689
**HSA-MIR-106B-5P**	18	6	7	410	5	-0,226787186
**HSA-MIR-5699-3P**	3	8	6	142	1	-0,226818978
**HSA-MIR-195-5P**	302	250	197	65	466	-0,232383069
**HSA-MIR-222-3P**	176	343	504	11176	1	-0,232490936
**HSA-MIR-4502**	1	22	19	413	1	-0,232991564
**HSA-MIR-940**	1	2	3	12	1	-0,234282072
**HSA-MIR-127-3P**	210	258	24	26324	27	-0,238966642
**HSA-MIR-23A-3P**	227	189	73	25191	14	-0,239109142
**HSA-MIR-22-5P**	3	2	1	240	1	-0,23923642

**HSA-MIR-548T-5P**	188	48	97	1	256	-0,239681862
**HSA-LET-7A-5P**	1159	2496	576	178632	692	-0,241020732
**HSA-MIR-18A-5P**	4	3	6	792	1	-0,244119131
**HSA-MIR-379-5P**	21	37	5	15790	2	-0,244894051
**HSA-MIR-671-5P**	16	5	2	6707	1	-0,24496236
**HSA-MIR-942-5P**	3	5	2	888	3	-0,245443167
**HSA-MIR-494-3P**	6	2	1	3225	2	-0,24567754
**HSA-LET-7B-5P**	48	177	74	31721	75	-0,245763253
**HSA-MIR-125B-1-3P**	8	2	13	987	4	-0,245894448
**HSA-MIR-26B-5P**	47	11	68	15818	17	-0,245950771
**HSA-MIR-329-3P**	1	2	2	786	1	-0,246164792
**HSA-MIR-1296-5P**	1	6	1	255	4	-0,24666385
**HSA-MIR-30D-5P**	4	2	52	12768	11	-0,247553152
**HSA-MIR-548H-5P**	4	6	1	166	6	-0,247802772
**HSA-MIR-193B-3P**	7	22	8	54	14	-0,248035157
**HSA-MIR-584-5P**	1	2	9	1821	7	-0,249576796
**HSA-MIR-484**	11	8	19	904	12	-0,249766128
**HSA-MIR-296-3P**	88	183	85	866	136	-0,249820161
**HSA-MIR-21-3P**	3	3	5	891	7	-0,25004405
**HSA-MIR-25-5P**	27	16	12	708	28	-0,250083933
**HSA-MIR-194-5P**	1	2	4	777	6	-0,251688013
**HSA-MIR-146A-3P**	56465	503	258	46	79174	-0,253138129
**HSA-MIR-767-3P**	1	2	5	28	1	-0,253190488
**HSA-MIR-335-3P**	11	11	8	356	16	-0,25592062
**HSA-MIR-1262**	295	3523	1082	102	3375	-0,256340316
**HSA-MIR-323A-3P**	1	9	10	1345	22	-0,257544182
**HSA-MIR-3065-3P**	6404	8329	4680	121	13630	-0,257909208
**HSA-MIR-99B-3P**	1	2	8	261	4	-0,259623453
**HSA-MIR-501-5P**	71	78	47	6	136	-0,263490288
**HSA-MIR-126-3P**	1	2	969	13346	98	-0,263521586
**HSA-MIR-15A-5P**	10	3	7	1452	40	-0,264808073

**HSA-MIR-193A-3P**	11	37	87	6	44	-0,265989641
**HSA-MIR-183-5P**	76	96	268	8269	268	-0,268353812
**HSA-MIR-136-3P**	7	11	20	735	28	-0,270511857
**HSA-MIR-625-3P**	37	31	55	4221	157	-0,270856932
**HSA-MIR-136-5P**	72	6	81	1139	75	-0,271700457
**HSA-MIR-215-5P**	6	3	2	52	7	-0,276855324
**HSA-MIR-548E-3P**	14	17	20	35	15	-0,277289982
**HSA-MIR-1287-5P**	6	9	14	143	12	-0,283969606
**HSA-MIR-3929**	7	6	1	54	10	-0,285813119
**HSA-MIR-181A-2-3P**	13	16	15	456	36	-0,287585491
**HSA-MIR-548D-5P**	24	26	6	17	41	-0,28910132
**HSA-MIR-1305**	28	2	22	83	22	-0,290227492
**HSA-MIR-582-3P**	3	12	11	34	8	-0,29688993
**HSA-MIR-937-3P**	148	453	505	21	524	-0,299323698
**HSA-MIR-143-3P**	1	2	1	35	4	-0,299332336
**HSA-MIR-4326**	3	5	22	647	42	-0,299854935
**HSA-MIR-3681-5P**	1389	974	632	2	2581	-0,30176347
**HSA-MIR-29C-5P**	13031	4426	2657	87	21905	-0,303756271
**HSA-MIR-3662**	3458	2473	2383	72	6163	-0,304564156
**HSA-MIR-708-3P**	699	1210	2253	6	1838	-0,306656944
**HSA-MIR-409-3P**	672	985	2414	12717	1463	-0,314529238
**HSA-MIR-561-3P**	18	43	5	32	47	-0,317118253
**HSA-MIR-485-5P**	21	19	4	189	39	-0,317709897
**HSA-MIR-31-3P**	150	304	178	319	250	-0,319895667
**HSA-MIR-139-3P**	1	2	3	65	7	-0,322296257
**HSA-MIR-30E-3P**	23	43	35	652	89	-0,324706509
**HSA-MIR-3128**	1	2	1	8	2	-0,333646724
**HSA-MIR-4445-3P**	14	16	4843	5	1591	-0,350529614
**HSA-MIR-376A-2-5P**	152917	474425	284492	108	636197	-0,352298365
**HSA-MIR-29A-5P**	11	31	25	350	63	-0,357677793
**HSA-MIR-708-5P**	4	2	3	26	7	-0,362867268

**HSA-MIR-939-5P**	4	2	3	16	5	-0,37281813
**HSA-MIR-19B-1-5P**	34353	44829	28019	21	89064	-0,376609439
**HSA-MIR-3183**	17	25	44	6	40	-0,377184138
**HSA-MIR-4653-3P**	913	748	1688	3	1880	-0,379244261
**HSA-MIR-2682-5P**	7	12	19	59	15	-0,379751782
**HSA-MIR-3925-5P**	1	3	3	9	3	-0,395084656
**HSA-MIR-6816-5P**	500	1008	502	6	1729	-0,396755843
**HSA-MIR-155-5P**	128	2592	222	46835	9849	-0,398045761
**HSA-MIR-657**	10	6	23	28	11	-0,403491313
**HSA-MIR-3916**	24	23	23	2	54	-0,418240136
**HSA-MIR-4286**	6	3	6	52	15	-0,428432678
**HSA-MIR-579-5P**	11932	14481	21748	16	31089	-0,434387893
**HSA-MIR-4430**	7024	70	31	2	15300	-0,445183303
**HSA-MIR-5584-3P**	47	28	29	1	107	-0,450956097
**HSA-MIR-6888-3P**	906	636	779	2	2110	-0,453857698
**HSA-MIR-502-3P**	14387	135	110	46	33077	-0,464677174
**HSA-MIR-370-5P**	1494	1686	2500	59	4028	-0,477507036
**HSA-MIR-6511B-3P**	1533	5003	7037	21	8689	-0,494173127
**HSA-MIR-629-3P**	25	307	98	58	439	-0,507886664
**HSA-MIR-212-3P**	4	5	8	17	8	-0,512895804
**HSA-MIR-105-5P**	4385	3489	3440	695	11616	-0,521969483
**HSA-MIR-212-5P**	65	130	25	19	316	-0,525957178
**HSA-MIR-4417**	1	5	3	2	5	-0,532539954
**HSA-MIR-5090**	3	5	1	5	8	-0,532636833
**HSA-MIR-29A-3P**	107352	418838	138038	66109	771298	-0,534364996
**HSA-MIR-4798-3P**	7	6	9	3	14	-0,535404414
**HSA-MIR-548AY-3P**	342	88	1	6	1097	-0,537079663
**HSA-MIR-5695**	140	65	85	7	384	-0,537797068
**HSA-MIR-340-3P**	6	2	23	25	11	-0,54026047
**HSA-MIR-4804-3P**	424	158	253	6	1170	-0,541377753
**HSA-MIR-486-5P**	3089	5248	8253	61	12682	-0,553408951

**HSA-MIR-6755-5P**	13	36	19	1	81	-0,556774257
**HSA-MIR-3126-3P**	692	1525	1342	6	3760	-0,577964261
**HSA-MIR-1471**	6	6	10	1	18	-0,580438663
**HSA-MIR-150-3P**	978	3455	3976	5	7273	-0,580750156
**HSA-MIR-2355-5P**	219	397	93	19	1362	-0,585322051
**HSA-MIR-636**	37858	43933	54077	22	149384	-0,587384108
**HSA-MIR-495-3P**	32245	562	31188	172	91065	-0,596609858
**HSA-MIR-219B-5P**	8043	8928	2030	19	52843	-0,624503195
**HSA-MIR-5009-5P**	51	47	28	5	261	-0,625033045
**HSA-MIR-1908-5P**	1	2	2	9	5	-0,631535575
**HSA-MIR-5095**	41	172	81	101	243	-0,632554802
**HSA-MIR-6785-5P**	5837	4561	14640	85	23660	-0,632556457
**HSA-MIR-4317**	8	9	8	1	46	-0,637950762
**HSA-MIR-181C-5P**	17	157	15	20	480	-0,638648341
**HSA-MIR-6758-5P**	198	133	530	2	831	-0,642813419
**HSA-MIR-4783-3P**	3	3	1	5	8	-0,645457032
**HSA-MIR-369-5P**	9212	34742	19104	1253	117003	-0,654842308
**HSA-MIR-3198**	775	948	1280	6	4347	-0,654964564
**HSA-MIR-590-5P**	12848	228	135	45	80879	-0,659152887
**HSA-MIR-377-5P**	4	29	60	61	39	-0,659499587
**HSA-MIR-889-3P**	8	17	8	343	207	-0,665886961
**HSA-MIR-6806-5P**	4	2	8	1	15	-0,672776875
**HSA-MIR-181C-3P**	14013	11102	12379	61	98107	-0,674556308
**HSA-MIR-16-1-3P**	10	185	22	20	721	-0,674724322
**HSA-MIR-571**	1	2	1	1	9	-0,675181805
**HSA-MIR-6780B-3P**	395	645	1517	2	2741	-0,678701567
**HSA-MIR-301B-3P**	258	264	307	53	1619	-0,683258764
**HSA-MIR-4644**	3	2	1	7	7	-0,686809481
**HSA-MIR-7155-5P**	4909	2890	7185	2	28528	-0,687048344
**HSA-MIR-585-5P**	4	2	5	1	22	-0,697819482
**HSA-LET-7B-3P**	137	5	1	43	1184	-0,700297694

**HSA-MIR-324-3P**	654	166	683	339	1942	-0,702313626
**HSA-MIR-149-5P**	89	354	307	1269	832	-0,70881572
**HSA-MIR-29B-3P**	30928	161599	26223	55120	918626	-0,712942082
**HSA-MIR-4429**	18	6	26	4	100	-0,714145208
**HSA-MIR-205-5P**	21	12	43	9	85	-0,717574911
**HSA-MIR-1285-5P**	7497	17765	13449	58	170126	-0,718463121
**HSA-MIR-1273F**	62	81	85	5	1060	-0,721922015
**HSA-MIR-3679-5P**	88	74	69	72	496	-0,724415731
**HSA-MIR-6850-3P**	7	5	8	3	62	-0,730340306
**HSA-MIR-935**	384	557	810	367	1178	-0,730475451
**HSA-MIR-3651**	24	64	86	6	392	-0,732842773
**HSA-MIR-330-5P**	17	12	8	430	331	-0,737039408
**HSA-MIR-183-3P**	319	579	783	58	7261	-0,737144937
**HSA-MIR-3615**	120	76	145	787	596	-0,745263494
**HSA-MIR-181D-5P**	631	2606	3634	102	35959	-0,74752997
**HSA-MIR-7847-3P**	4	3	10	1	97	-0,74920353
**HSA-MIR-1322**	32	48	151	8	349	-0,749273724
**HSA-MIR-576-3P**	357	1074	2715	99	29984	-0,757426528
**HSA-MIR-24-2-5P**	10	9	22	8	213	-0,75845881
**HSA-MIR-1228-3P**	3	5	2	7	10	-0,759454526
**HSA-MIR-26A-2-3P**	31	20	41	36	263	-0,777916528
**HSA-MIR-450A-5P**	6	2	1	40	371	-0,783460945
**HSA-MIR-3657**	11	5	60	9	136	-0,797407962
**HSA-MIR-3187-3P**	38	129	890	60	2297	-0,800774411
**HSA-MIR-5000-3P**	11	2	64	26	70	-0,819050002
**HSA-MIR-548U**	1	3	1	11	16	-0,825139659
**HSA-MIR-483-5P**	7	5	11	12	37	-0,856777224
**HSA-MIR-3064-5P**	7	8	67	25	87	-0,856836984
**HSA-MIR-190A-5P**	176	285	211	1876	4242	-0,864580982
**HSA-MIR-93-5P**	6760	18488	18175	28622	80353	-0,872233826
**HSA-MIR-664B-5P**	7	3	10	12	23	-0,894863354

**HSA-MIR-410-5P**	14	31	28	59	117	-0,896601184
**HSA-MIR-200C-3P**	182	56	528	387	1066	-0,908852471
**HSA-MIR-641**	20	40	78	85	249	-0,909503842
**HSA-MIR-186-5P**	11	74	202	625	1161	-0,931155823
**HSA-MIR-125B-5P**	1391	2045	3818	5615	12236	-0,933677607
**HSA-MIR-1301-3P**	124	76	236	406	537	-0,945618054
**HSA-MIR-7-5P**	135	199	370	586	692	-0,971416091

1. Love MI, Huber W and Anders S: Moderated estimation of fold change and dispersion for RNA-seq data with DESeq2. Genome Biol 15: 550, 2014.

Appendix S1. Results of small tandem repeat (STR) profiling of HT20 cell line

GENDER	X , X
D3S1358	16 , 16
TH01	7 , 9.3
D12S391	17 , 18
D5S818	12 , 12
TPOX	11 , 11
D2S441	13 , 13
D7S820	10, 10
D13S317	11 , 11
FGA	-
D22S1045	15 , 16
D18S51	13 , 16
D16S539	11 , 14
D8S1179	13 , 14
CSF1PO	12 , 12
D6S1043	14 , 14
vWA	16 , 17
D21S11	28 , 32.2
SE33	23.2 , 25.2
D10S1248	14 , 14
D1S1656	14 , 17
D19S433	12 , 12
D2S1338	19 , 24
DYS391	9
Yindel	2

Appendix S2. Results of small tandem repeat (STR) profiling of HBL100 cell line

GENDER	X , Y
D3S1358	14 , 16
TH01	6 , 8
D12S391	18 , 18
D5S818	11 , 12
TPOX	8 , 8
D2S441	11 , 11
D7S820	8 , 12
D13S317	12 , 12
FGA	25 , 25
D22S1045	15 , 16
D18S51	16 , 16
D16S539	9 , 12
D8S1179	12 , 15
CSF1PO	10 , 10
D6S1043	12 , 14
vWA	16 , 16
D21S11	28 , 28
SE33	27.2 , 29.2
D10S1248	13 , 13
D1S1656	12 , 16.3
D19S433	15 , 15
D2S1338	18 , 24
DYS391	11
Yindel	2

Appendix S3. Results of small tandem repeat (STR) profiling of MCF7 cell line

GENDER	X , X
D3S1358	14 , 16
TH01	6 , 6
D12S391	-
D5S818	11 , 12
TPOX	9 , 12
D2S441	11 , 14
D7S820	8 , 9
D13S317	11 , 11
FGA	21 , 21
D22S1045	15 , 15
D18S51	13 , 17
D16S539	11, 12
D8S1179	13 , 13
CSF1PO	10 , 10
D6S1043	11 , 11
vWA	14, 15
D21S11	30 , 30
SE33	15 , 22
D10S1248	14 , 14
D1S1656	11 , 12
D19S433	14 , 14
D2S1338	19 , 24
DYS391	-
Yindel	-

Appendix S4. Results of small tandem repeat (STR) profiling of MDAMB231 cell line

GENDER	X , X
D3S1358	16 , 16
TH01	7 , 9.3
D12S391	17 , 18
D5S818	12 , 12
TPOX	8 , 9
D2S441	14 , 15
D7S820	8 , 9
D13S317	13 , 13
FGA	22 , 23
D22S1045	16 , 16
D18S51	11 , 16
D16S539	12 , 12
D8S1179	13 , 13
CSF1PO	12 , 13
D6S1043	18 , 18
vWA	15 , 18
D21S11	30 , 33.2
SE33	15 , 15
D10S1248	14 , 16
D1S1656	15 , 17
D19S433	11 , 14
D2S1338	20 , 21
DYS391	-
Yindel	-

Appendix S5. Results of small tandem repeat (STR) profiling of MDAMB453 cell line

GENDER	X , X
D3S1358	15 , 15
TH01	6 , 6
D12S391	18 , 21
D5S818	11 , 11
TPOX	10 , 10
D2S441	10 , 10
D7S820	10 , 10
D13S317	12 , 12
FGA	18 , 23
D22S1045	15 , 16
D18S51	15 , 20
D16S539	9 , 9
D8S1179	10 , 12
CSF1PO	10 , 12
D6S1043	11 , 18
vWA	17 , 18
D21S11	29 , 31
SE33	24.2 , 28.2
D10S1248	14 , 15
D1S1656	16.3 , 17.3
D19S433	13 , 14
D2S1338	23 , 24
DYS391	-
Yindel	-
